# Enterocyte-Derived and Catalytically Active Transglutaminase 2 in the Gut Lumen of Mice: Implications for Celiac Disease

**DOI:** 10.1053/j.gastro.2024.05.029

**Published:** 2024-05-31

**Authors:** MAUREEN T. MELING, LIV KLEPPA, HARRISON A. BESSER, CHAITAN KHOSLA, M. FLEUR DU PRÉ, LUDVIG M. SOLLID

**Affiliations:** Norwegian Coeliac Disease Research Centre, Institute of Clinical Medicine, University of Oslo, Oslo, Norway; Department of Immunology, Oslo University Hospital, Oslo, Norway; Norwegian Coeliac Disease Research Centre, Institute of Clinical Medicine, University of Oslo, Oslo, Norway; Department of Immunology, Oslo University Hospital, Oslo, Norway; Department of Chemistry, Stanford University, Stanford, California; Stanford Medical Scientist Training Program, Stanford University School of Medicine, Stanford, California; Department of Chemistry, Stanford University, Stanford, California; Department of Chemical Engineering, Stanford University, Stanford, California; Sarafan ChEM-H, Stanford University, Stanford, California; Norwegian Coeliac Disease Research Centre, Institute of Clinical Medicine, University of Oslo, Oslo, Norway; Department of Immunology, Oslo University Hospital, Oslo, Norway; Norwegian Coeliac Disease Research Centre, Institute of Clinical Medicine, University of Oslo, Oslo, Norway; Department of Immunology, Oslo University Hospital, Oslo, Norway

Celiac disease is an autoimmune enteropathy caused by harmful immune reactions to cereal gluten proteins.^1^ The enzyme transglutaminase 2 (TG2) is involved in the pathogenesis both by being the target of highly disease-specific autoantibodies and by deamidating gluten peptides, thereby allowing their presentation to T cells by HLA-DQ2 or HLA-DQ8 molecules. The autoantibodies to TG2 are only produced in individuals expressing HLA-DQ2/-DQ8 individuals who consume gluten. This HLA- and gluten-dependence of TG2 autoantibodies can be explained by a hapten carrier–like model where, by involvement of covalent gluten peptide-TG2 complexes taken up TG2-specific B cells, gluten-specific CD4^+^ T cells can provide help to TG2-specific B cells.

TG2-specific plasma cells, being effector B cells, are abundant in celiac disease small intestinal lesions.^1^ Small intestinal lamina propria plasma cells are generated after interactions between T cells and B cells in gut-associated lymphoid tissue, and their specificities reflect antigens of the gut lumen, such as bacteria and food,^2^ suggesting that luminal TG2 is a priming factor in the history of these plasma cells. Further, based on the observations that (1) 2 × 10^10^ epithelial cells are shed into the gut lumen in humans every day,^3^ (2) B cells are likely not exposed to TG2 during their development,^4^ and (3) TG2 is abundantly expressed in enterocytes,^5^ a previous study suggested that pathogenic TG2 is a luminal enzyme derived from shed enterocytes.^5^

This previous study demonstrated expression of catalytically active TG2 released from freeze-thawed mouse enterocytes and showed that TG2-reactive, but not TG2 nonreactive B-cell transfectants, can present deamidated gluten epitopes to T cells.^5^ The questions remained, however, whether catalytically active TG2 exists in gut lumen and, if so, whether the main source is indeed enterocytes. This study aimed to address both of these issues.

We first established an assay combining magnetic bead-based immunoprecipitation of TG2 and Western blot protein visualization that is capable of detecting TG2 in mouse gut lavage fluids. We observed that recombinant mouse TG2 spiked into gut lavage fluid of mice could be detected if protease inhibitors, specifically soybean trypsin inhibitor and Pefabloc (Roche), were added to the lavage fluid (Figure 1A). This indicates that TG2 is prone to rapid proteolytic degradation in the mouse gut lumen, but given that celiac disease antibodies detect conformational epitopes of TG2,^1^ it is conceivable that B cells in gut-associated lymphoid tissue can bind the structurally intact enzyme.

We next performed gut luminal lavage on the small intestine and used the established TG2 detection assay to address whether endogenous TG2 is present in luminal fluid of mice. Although TG2 could be detected in luminal fluid of *Tgm2* wild-type (WT) C57BL/6 mice in the presence of protease inhibitors, the protein could not be detected in luminal fluid of *Tgm2* knockout (TG2^KO^) mice (Figure 1B). Luminal TG2 could be detected in all segments of the small intestine (Supplementary Figure 1A). Reassuringly, an analysis of lavage fluids of newly generated intestinal epithelial cell (IEC)-specific TG2-KO (TG2^IEC-KO^) mice (Supplementary Figure 1B) could not detect TG2 (Figure 1B).

To investigate luminal TG2 expression under an inflammatory condition, WT and TG2^IEC-KO^ mice were treated with interferon-gamma (IFN-*γ*), a cytokine that induces an enteropathy with tissue-remodeling features similar to celiac disease,^6^ and thus potentially reflecting increased TG2 expression due to higher cell turnover or increased amount per cell. Luminal TG2 levels were higher in WT mice treated with IFN-*γ* compared with sham-treated mice (*P* < .005), whereas for TG2^IEC-KO^, no luminal TG2 was detected regardless of treatment (Figure 1C). Furthermore, we observed no TG2 in the EDTA/dithiothreitol stripped epithelial layer of TG2^IEC-KO^ mice even after IFN-*γ* treatment, suggesting that intraepithelial leukocytes contribute negligibly to luminal TG2 (Supplementary Figure 1C).

To test for catalytic activity of luminal TG2, we used the newly developed sulfo-Cy5-labeled irreversible TG2 inhibitor HB-230 and its negative control analog HB-258.^7^ HB-230 is a gluten peptide mimetic based on the TG2 substrate sequence PQLPY. HB-258 is identical to HB-230 except it lacks the electrophilic warhead that allows irreversible binding to the active site cysteine. When adding these compounds to the lavage fluid of WT mice in the presence of protease inhibitors, we observed that HB-230 but not HB-258 bound to TG2, indicating that luminal TG2 is indeed catalytically active (Figure 1D).

In addition, upon administration of native gluten peptides in ligated loops of the small intestine of live mice and using a celiac patient-derived monoclonal antibody specific for a deamidated gluten peptide, we found deamidation in WT but not in KO mice (*P* < .05), confirming the ability of luminal TG2 to deamidate gluten peptides (Figure 1E and Supplementary Figure 1D). Of note, no Ca^2+^ was added in these experiments, indicating that the calcium concentration in this environment is sufficient to render TG2 active.

In line with earlier observations,^8^ most of the shed cells obtained from ligated loops were epithelial cell adhesion molecule (EpCAM)^+^/CD45^−^ epithelial cells (Supplementary Figure 1E). A considerable fraction of the enterocyte was viable, suggesting that such cells can release cytosolic TG2 into the lumen upon disintegration (Supplementary Figure 1F and Supplementary Table 1).

The findings demonstrate the presence of live enterocytes within the small intestinal gut lumen and in vivo release of catalytically active TG2 into the lumen of mice. A single-cell sequencing study of mice also concluded that many luminal epithelial cells are viable, and this study also reported that the TG2 messenger RNA is higher in epithelial cells in the lumen than in the villi.^9^ Collectively, the observations of viable epithelial cells in the gut lumen challenge the notion that the gut epithelial cells shed as apoptotic cells or undergo rapid anoikis after shedding.^10^ The large biomass of shed gut epithelial cells undergoing disruption in the gut lumen conceivably has implications that go beyond TG2 and celiac disease, possibly affecting many aspects of gut physiology.

Our study provides further evidence that enterocyte-derived TG2 is pathogenic in celiac disease. Luminal TG2 by mediating formation of free deamidated gluten peptides and by engaging in formation of TG2-gluten may lead to activation of gluten-specific T cells and TG2-specific B cells. Hence, inhibition of luminal TG2 activity is a credible therapeutic approach in celiac disease.

## Supplementary Material

Supplementary **Supplementary Table 1.** Characterization of Shed Cells Shed in a 20-cm Ligated Small Intestine Loop by Flow Cytometry

Supplementary figure **Supplementary Figure 1.** Luminal TG2 could be detected in all segments of the small intestine and the source is mouse enterocytes. (*A*) The small intestine of WT mice was equally divided into 3 parts (proximal, middle, and distal), and intestinal lavage containing protease inhibitor mixture was performed. Then, luminal TG2 was captured by immunoprecipitation and Western blotting. Control: 12 nmol/L recombinant mouse (rm)TG2 in Tris-buffered saline (TBS). (*B*) Epithelial lysates from WT, TG2^IEC-KO^, and TG2^KO^ showing that only the WT lysate contained TG2, with no differences in the expression of villin1. (*C*) The epithelial cell layer of TG2^IEC-KO^ mice was separated from the lamina propria by use of EDTA and dithiothreitol. TG2 expression in lysates of stripped cells and remaining tissue was compared between IFN-*γ*– and sham-treated mice by Western blot. *β*-Actin was detected as the reference protein, and rmTG2 (6 nmol/L) was used as the control. Note that no TG2 was detected in the stripped cells even after IFN-*γ* treatment, whereas the protein was detected in the remaining mucosal tissue of TG2^IEC-KO^ mice. (*D*) Specificity of the ELISA based on human immunoglobulin G1 anti-DGP antibody UCD1002–1E03 used to detect deamidated peptide was confirmed by serial dilutions of native (biotin-GSGSGS-PLQPQQPFP) and deamidated (biotin-GSGSGS-PLQPEQPFP) gluten peptides. (*E*) Detection of shed cells in the gut lumen. Small-intestinal luminal content was collected in a ligated intestinal loop assay during 30 minutes, and cells were collected by flushing intestinal loops with phosphate-buffered saline/2% fetal calf serum. Enterocytes were identified as EpCAM^+^/CD45^−^ and leukocytes as EpCAM^−^/CD45^+^ cells. Live cells were defined as 7-aminoactinomycin D (7-AAD)^−^/annexin V^−^, early apoptotic cells as 7-AAD^−^/annexin V^+^, and dead cells as 7-AAD^+^/annexin V^+^. EpCAM, epithelial cell adhesion molecule; FSC-A, forward scatter area; SSC-A, side scatter area. (*F*) Quantification of shed enterocytes with results from 6 mice. Box and whisker plot: The *boxes* indicate the 25th percentile (*bottom border*), median (*center line*), and 75th percentile (*top border*), and the *whiskers* show the maximum and minimum ranges.

## Figures and Tables

**Figure 1.**
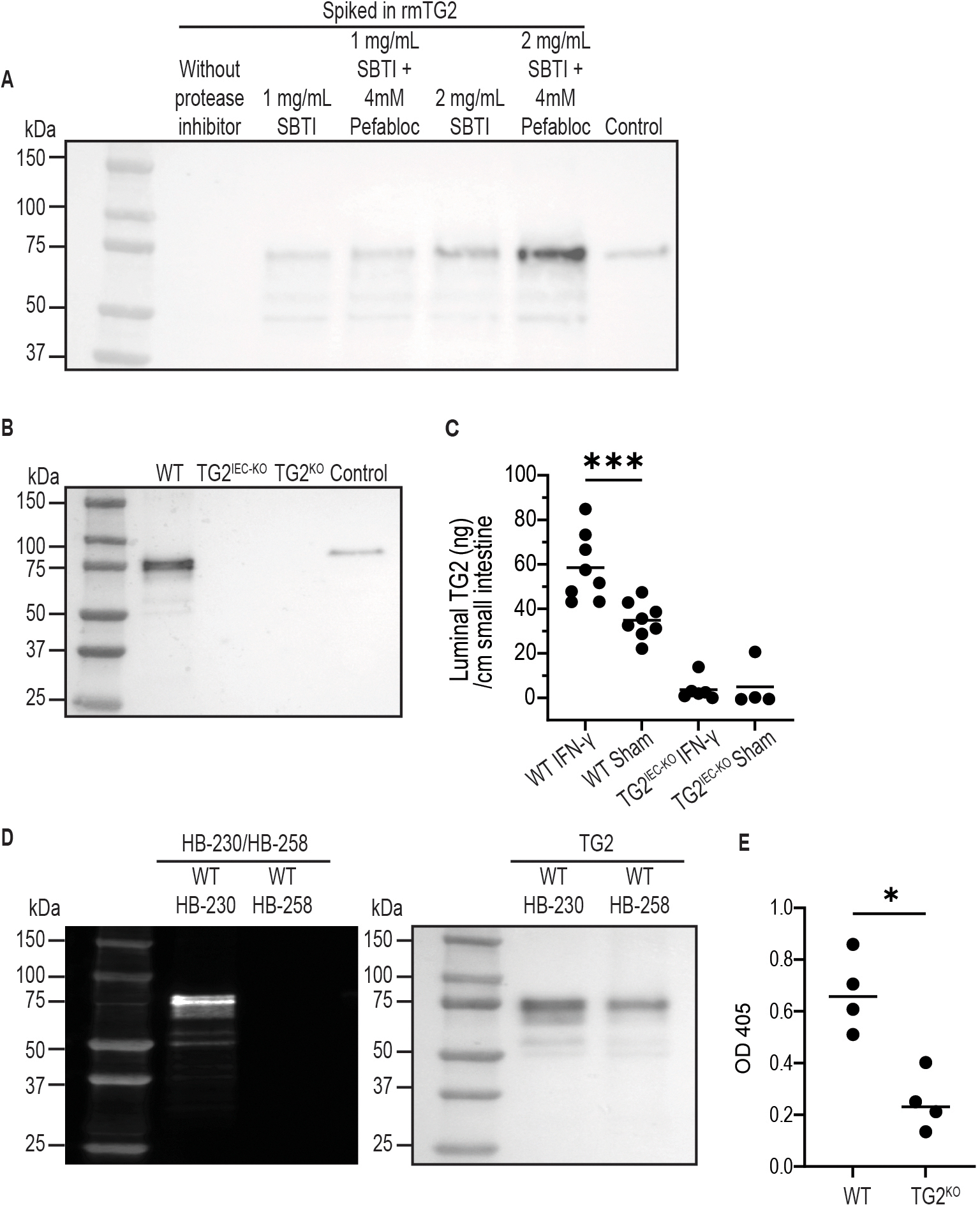
TG2 identified within the mouse intestinal lumen originates from shed enterocytes. (*A*) Small intestinal lavage fluid from *Tgm2* WT mice was collected using phosphate-buffered saline (PBS) or PBS supplemented with indicated concentrations of soybean trypsin inhibitor (SBTI) and Pefabloc (Roche). Lavage fluid was spiked with 130 nmol/L of recombinant mouse (rm) TG2, and TG2 was detected by Western blot. Control lane: 12 nmol/L rmTG2 in Tris-buffered saline (TBS). (*B*) Western blot demonstrating presence of endogenous luminal TG2, which was extracted from mice small intestinal lavage fluid via immunoprecipitation. The analysis included samples from *Tgm2* WT, TG2^IEC-KO^, and *Tgm2* KO (TG2^KO^) mice. Control lane: 12 nmol/L rmTG2 in TBS. (*C*) Luminal TG2 was quantified using enzyme-linked immunosorbent assay (ELISA). The amount of luminal TG2 in IFN-*γ*–treated WT mice (n = 8) was significantly higher compared with sham-treated WT mice (n = 8). ***P < .005, Mann-Whitney test. No significant differences were observed between IFN-*γ*–treated TG2^IEC-KO^ mice (n = 6) and sham-treated TG2^IEC-KO^ mice (n = 4) (*P* > .05, Mann-Whitney test). (*D*) Small intestinal lavage fluid from WT mice was incubated with 1 *μ*mol/L of the irreversible TG2 inhibitor HB-230 or 1 *μ*mol/L of the negative control compound HB-258 to detect TG2 catalytic activity. Cy5 fluorescence reports on incorporation of HB-230 or HB-258. (*E*) Deamidation of a biotinylated *γ*−/*ω*-gliadin nonapeptide within the gut lumen was quantified using ELISA, with results reported as optical density (OD) units. The levels of deamidated gluten peptide (DGP) in WT mice (n = 4) were significantly higher compared with those observed in TG2^KO^ mice (n = 4) (*P* < .05, Mann-Whitney test).

## Data Availability

Data, methods, and biological materials used to conduct the research of this paper for purposes of reproducing the results or replicating the procedure will be made available upon reasonable request.

## Abbreviations used in this paper:

IFN-*γ*: interferon gamma
IEC: intestinal epithelial cell
TG2: transglutaminase 2
TG2^IEC-KO^: intestinal epithelial cell specific *Tgm2* knockout
TG2^KO^: *Tgm2* knockout
WT: wild-type
