# Supplementary material for: Enterocyte-Derived and Catalytically Active Transglutaminase 2 in the Gut Lumen of Mice: Implications for Celiac Disease
Source: Gastroenterology. Author manuscript; Available in PMC 2025 Oct 1. (PMC12087371; doi:10.1053/j.gastro.2024.05.029)
Supplement: Supplementary — Table 1. Characterization of Shed Cells Shed in a 20-cm Ligated Small Intestine Loop by Flow Cytometry [file NIHMS2077461-supplement-Supplementary.pdf]

Supplemental Material

Enterocyte-derived and catalytically active transglutaminase 2 in the gut lumen of mice: Implications for celiac disease

Maureen T. Meling[1,2], Liv Kleppa[1,2], Harrison A. Besser[3,4], Chaitan Khosla[3,5,6], M. Fleur du Pré[1,2] and Ludvig M. Sollid[1,2]

[1]Norwegian Coeliac Disease Research Centre, Institute of Clinical Medicine, University of Oslo, Oslo, Norway

[2]Department of Immunology, Oslo University Hospital, Oslo, Norway

[3]Department of Chemistry, Stanford University, Stanford, California, United States

[4]Stanford Medical Scientist Training Program, Stanford University School of Medicine, Stanford, California, United States

[5]Department of Chemical Engineering, Stanford University, Stanford, California, United States

[6]Sarafan ChEM-H, Stanford University, Stanford, California, United States

Supplementary Methods

Mice and cytokine injections

To generate intestinal epithelial cell (IEC) specific *Tgm2* knockout ($TG2^{IEC-KO}$) mice, *Tgm2*$^{flox/flox}$ mice[1] (stock #024694, The Jackson Laboratory) were crossed with heterozygous villin-cre (*vil1*-cre) transgenic mice[2] (stock #021504, The Jackson Laboratory), both on C57Bl/6 background. From the filial (F) 1 progeny, *Tgm2*$^{flox/wt}$ *Vil1*-cre transgenic mice were bred to *Tgm2*$^{flox/flox}$ mice to generate *Tgm2*$^{flox/flox}$ *Vil1*-cre transgenic $TG2^{IEC-KO}$ mice. Full *Tgm2* knockout ($TG2^{KO}$)[3] and *Tgm2* wild-type (WT) mice on C57Bl/6 background were bred in-house or purchased from Janvier-Labs. Male and female mice were included in experiments at 8 to 16 weeks of age. Mice were fed with a gluten-free open standard diet with 15 kcal% fat (D11112201i, Research Diets). Mice were injected intraperitoneally with 50 µg mouse interferon-γ (GenScript) dissolved in 600 µl PBS daily, or sham (PBS) only, divided over 3 equal doses every 8-hour interval for 3 days,[4] and euthanized 3 hours after the final injection. All experiments were approved by the Norwegian Food Safety Authority (Mattilsynet) and were conducted in accordance with Directive 2010/63/EU of the European Union.

Small intestinal lavage

The small intestines were clamped using hemostatic forceps and injected with 1 ml PBS using a feeding needle (AgnThos) and incubated for 10 min on ice. In some experiments, soybean trypsin inhibitor (SBTI, Sigma) and Pefabloc (Roche) in indicated concentrations were added. The intestinal content was collected and kept on ice until protein analysis. To analyze TG2 in different segments, the full-length small intestine was divided it into three equal parts: proximal, middle and distal.

Cell isolation and lysate preparation

To obtain IECs, small intestines were cleared from fat and mesentery, cut open longitudinally and flushed twice with ice-cold 10 mM HEPES/HBSS. The tissue was then cut into ~0.5-1 cm pieces and transferred to pre-warmed (37°C) HBSS containing 5 mM EDTA and 1 mM DTT. After incubation for 20 min at 37°C under constant rotation, the suspension was filtered through a 100 µm cell strainer (Miltenyi), and epithelial cell lysates were generated by two cycles of snap-freezing in liquid N_2 and thawing at room temperature, or using Pierce™ RIPA buffer supplemented with 1x Halt™ Protease Inhibitor Cocktail (ThermoScientific). To generate lysate of the small intestinal tissue that remains after stripping off the epithelial layer, the EDTA/DTT incubation as described above was repeated, and 20 mg remaining tissue was lysed using RIPA buffer.

Immunoprecipitation and Western blotting

Protein G Dynabeads (1.5 mg, Invitrogen) were incubated with 3 µg celiac recombinant monoclonal human IgG1 anti-TG2 antibody (679-14-E06)[5] in 200 µl TBS 0.02% Tween pH 7.4 for 10 min at RT. Unbound antibody was removed by magnetic separation and washing. The beads were added to 800 µl lavage fluid and incubated for 40 min at RT. Bound proteins were eluted using 2x Laemmli sample buffer followed by SDS-Page (4-20% TGX) and semidry transfer to a nitrocellulose membrane. The membranes were blocked using 5% (w/v) dry milk in TBS 0.1% Tween, and TG2 was detected using a custom rabbit-anti-TG2 polyclonal antibody (1:20.000, Pacific Immunology), followed by HRP-conjugated goat-anti-rabbit IgG (1:3000, Southern Biotech). Recombinant mouse TG2 (rmTG2) was expressed in *E. coli* as previously described.[6] To probe for TG2 activity, the irreversible TG2 inhibitor HB-230 and control compound HB-258, generated as previously described,[7] were incubated at 1 µM with small

intestinal lavage fluid prior to immunoprecipitation. Signals were developed by Supersignal West Pico PLUS chemiluminescent substrate (Thermo Scientific) and detected using a G:BOX chemiluminescence imaging system (Syngene) or ChemiDoc system (BioRad) for detection of the Cy5 fluorescent signal.

Lysates of isolated cells and remaining small intestinal tissue were incubated with rabbit-anti-mouse TG2 and rabbit-anti-villin1 polyclonal antibody (1:8000, Novus Biologicals) or rabbit-anti-β-actin antibody (1:3000, Cell Signaling Technology) followed by HRP-conjugated goat-anti-rabbit IgG.

Intestinal loop assay

Mice were anesthetized and a 3-5 cm incision was made to expose the small intestine. For the collection of shed cells, a ligated loop of ∼20 cm was made and injected with 0.5 ml of 2% FCS/PBS containing 2 mg/ml SBTI and 4 mM Pefabloc through a loose ligation roughly 3 cm below the stomach. The loop was tightened, the abdomen closed and mice were kept under anesthesia for 30 minutes. The ligated loop was removed and luminal content was collected with 2 ml cold 2% FCS/PBS.

To detect gluten peptide deamidation in the lumen, a ~6 cm ligated loop was injected with 300 µl of 100 µM biotinylated 9mer native γ-/ω-gliadin peptide (biotin-GSGSGS-PLQPQQPFP, GL Biochem)[8] in PBS/SBTI/Pefabloc. After 30 minutes, the ligated loop was removed and luminal content was collected with 200 µl of PBS/SBTI/Pefabloc. Samples were snap-frozen and stored -80^{0}C.

Flow cytometry

Flush fluid from the ligated loop was passed through a 100 µm strainer, centrifuged for 10 min at 350xg at 4°C, followed by staining with BV711-conjugated anti-mouse CD45 (clone 30-F11, 1:800, Biolegend) and eFluor450-conjugated anti-mouse CD326 (EpCAM, clone G8.8, 1:2000, Biolegend) for 20 minutes on ice in 2% FCS/PBS containing 2 mM EDTA. To distinguish between live, early apoptotic, and dead cells, 7-AAD (eBioscience) and PE-Cy7-Annexin V (Thermo Fisher) were added 10 min prior to acquisition on an Attune NxT flow cytometer (Thermo Fisher).

ELISA

For TG2 quantification, ELISA plates were coated with 2 µg/ml rabbit-anti-mouse TG2. After washing and blocking, 100 µl lavage fluid (duplicates) was incubated for 2h. Serial dilution of rmTG2 spiked in lavage fluid from $TG2^{KO}$ mice was used as standard curve. After washing, the plates were incubated with human IgG1 anti-TG2 antibody (679-14-E06) and detected with rabbit anti-human IgG-alkaline-phosphatase (1:2000, Abcam).

To detect deamidated gluten peptide (DGP) in ligated loop samples, 0.2 mM TG2 inhibitor DP3-3 (Ac-P(DON)LPF-NH2, Zedira) was added to prevent extraluminal deamidation and samples were thawed on ice. ELISA plates were coated with 6 µg/ml celiac patient derived recombinant monoclonal human IgG1 anti-DGP antibody (UCD1002-1E03)[8]. Biotinylated peptide was detected by alkaline-phosphatase-conjugated streptavidin (1:3000, Southern Biotech). Optical density was determined at 405 nm. A standard curve and ascertainment of no reactivity to native peptide was established by use of synthetic peptides (biotin-GSGSGS-PLQPQQPFP and biotin-GSGSGS-PLQPEQPFP, both GL Biochem)[8].

References

1. Nanda N, Iismaa SE, Owens WA, et al. J Biol Chem 2001;276:20673-8.
2. Madison BB, Dunbar L, Qiao XT, et al. J Biol Chem 2002;277:33275-83.
3. De Laurenzi V, Melino G. Mol Cell Biol 2001;21:148-55.
4. Eriguchi Y, Nakamura K, Yokoi Y, et al. JCI Insight 2018;3.
5. Di Niro R, Mesin L, Zheng NY, et al. Nat Med 2012;18:441-5.
6. **Iversen R, Di Niro R**, Stamnaes J, et al. J Immunol 2013;190:5981-91.
7. Loppinet E, Besser HA, Sewa AS, et al. Cell Chem Biol 2023;30:55-68.e10.
8. **Snir O, Chen X, Gidoni M**, et al. JCI Insight 2017;2.

		Mouse 1	Mouse 2	Mouse 3	Mouse 4	Mouse 5	Mouse 6	Median
EpCAM positive / CD45 negative cells								
(%)		19	39	21	35,2	18,3	31,7	26,4
	7-AAD negative / Annexin V negative cells							
	(%)	41,2	57,3	34,6	18,7	41,3	43,3	41,3
	7-AAD negative / Annexin V positive cells							
	(%)	18,7	21,3	24,4	45,7	27	41,1	25,7
	7-AAD positive / Annexin V positive cells							
	(%)	36,2	19	38,6	34,7	27,4	14,1	31,1
EpCAM negative / CD45 positive cells								
(%)		1,5	5	1,5	3	16,8	3	3,0
	7-AAD negative / Annexin V negative cells							
	(%)	54,6	77,2	55,6	76,4	76,2	41,4	65,9

7-AAD negative / Annexin V positive cells (%)	2,7	3,5	2	6,2	16,6	4,4	4,0
7-AAD positive / Annexin V positive cells (%)	37,4	15,7	26,2	12,7	5,6	30,4	21,0

Supplementary Table 1. Characterization of shed cells shed in a 20-cm ligated small intestine loop by flow cytometry. EpCAM, epithelial cell adhesion molecule; 7-AAD, 7-aminoactinomycin D.

Supplementary Figure 1. Luminal TG2 could be detected in all segments of the small intestine and the source is mouse enterocytes. (A) The small intestine of WT mice was equally divided into three parts (proximal, middle, and distal), which were then subjected to intestinal lavage containing protease inhibitor mixture. Then, luminal TG2 was captured by immunoprecipitation and Western blotting. Control:12 nM rmTG2 in TBS. **(B)** Epithelial lysates from WT, $TG2^{IEC-KO}$, and $TG2^{KO}$ showing that only the WT lysate contained TG2, with no differences in the expression of villin1. **(C)** The epithelial cell layer of $TG2^{IEC-KO}$ mice was separated from the lamina propria by use of EDTA and DTT. TG2 expression in lysates of stripped cells and remaining

tissue was compared between IFN-γ and sham treated mice by Western blot. β-actin was detected as the reference protein, and rmTG2 (6nM) was used as control. Note that no TG2 was detected in the stripped cells even after IFN-γ-treatment, while the protein was detected in the remaining mucosal tissue of $TG2^{IEC\text{-}KO}$ mice. **(D)** Specificity of the ELISA based on hIgG1 anti-DGP antibody UCD1002-1E03 used to detect deamidated peptide was confirmed by serial dilutions of native (biotin-GSGSGS-PLQPQQPFP) and deamidated (biotin-GSGSGS-PLQPEQPFP) gluten peptides. **(E)** Detection of shed cells in the gut lumen. Small intestinal luminal content was collected in a ligated intestinal loop assay during 30 minutes, and cells were collected by flushing intestinal loops with PBS/2% FCS. Enterocytes were identified as EpCAM+/CD45- and leukocytes as EpCAM-/CD45+ cells. Live cells were defined as 7-AAD-/Annexin V-, early apoptotic cells as 7-AAD-/Annexin V+ and dead cells as 7-AAD+/Annexin V+. **(F)** Quantification of shed enterocytes with results from six mice. 7-AAD, 7-aminoactinomycin D; DGP, deamidated gluten peptide; EpCAM, epithelial cell adhesion molecule; IFN-γ, interferon-γ ; rmTG2, recombinant mouse TG2; TG2, transglutaminase 2; $TG2^{IEC\text{-}KO}$, intestinal epithelial cell specific *Tgm2* knockout; TG2KO, *Tgm2* knockout; WT, wild-type.
