# Supplementary figures and images for: Enterocyte-Derived and Catalytically Active Transglutaminase 2 in the Gut Lumen of Mice: Implications for Celiac Disease
Source: Gastroenterology. Author manuscript; Available in PMC 2025 Oct 1. (PMC12087371; doi:10.1053/j.gastro.2024.05.029)

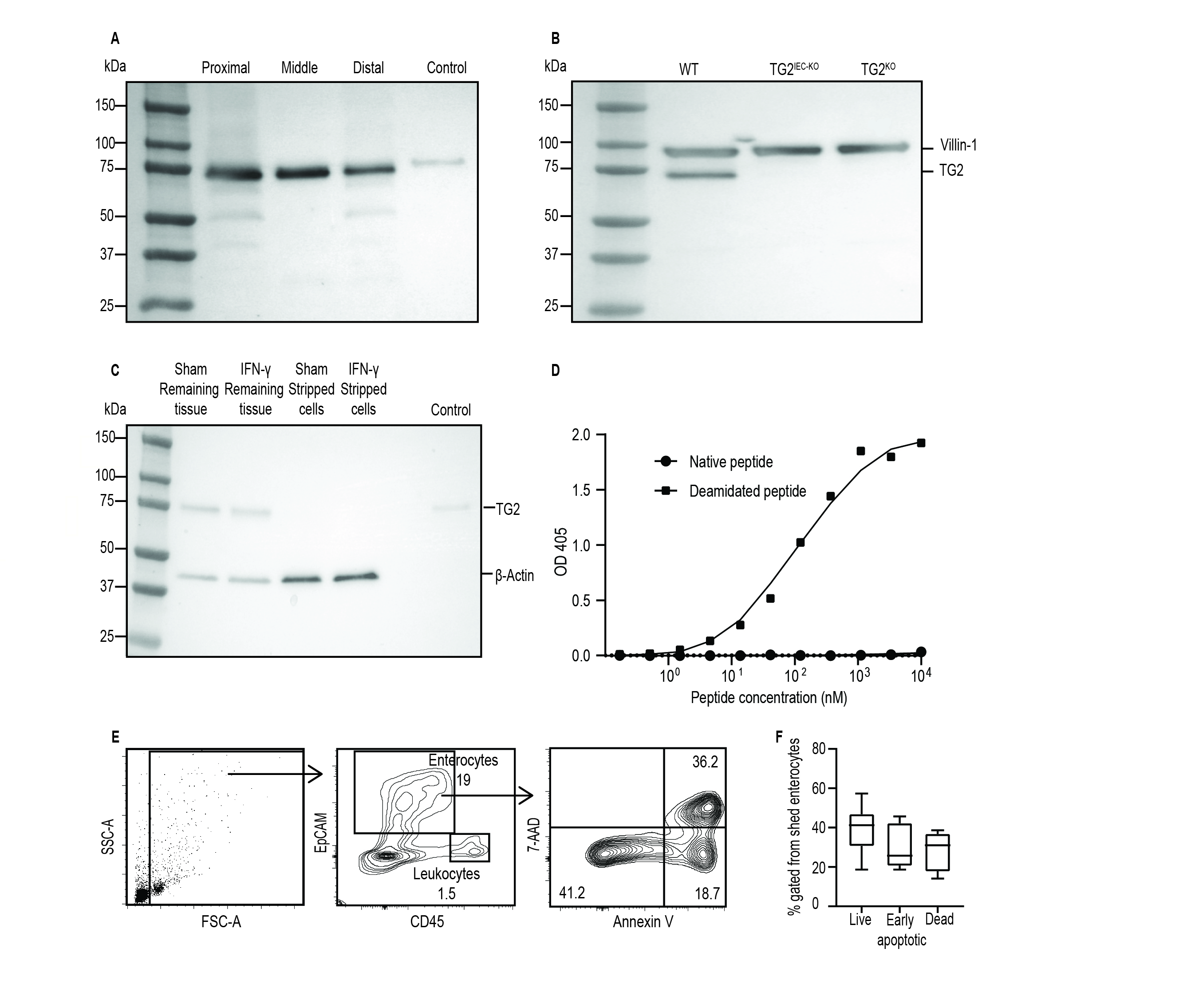

Supplement: Supplementary figure — 1. Luminal TG2 could be detected in all segments of the small intestine and the source is mouse enterocytes. (A) The small intestine of WT mice was equally divided into 3 parts (proximal, middle, and distal), and intestinal lavage containing protease inhibitor mixture was performed. Then, luminal TG2 was captured by immunoprecipitation and Western blotting. Control: 12 nmol/L recombinant mouse (rm)TG2 in Tris-buffered saline (TBS). (B) Epithelial lysates from WT, TG2IEC-KO, and TG2KO showing that only the WT lysate contained TG2, with no differences in the expression of villin1. (C) The epithelial cell layer of TG2IEC-KO mice was separated from the lamina propria by use of EDTA and dithiothreitol. TG2 expression in lysates of stripped cells and remaining tissue was compared between IFN-γ– and sham-treated mice by Western blot. β-Actin was detected as the reference protein, and rmTG2 (6 nmol/L) was used as the control. Note that no TG2 was detected in the stripped cells even after IFN-γ treatment, whereas the protein was detected in the remaining mucosal tissue of TG2IEC-KO mice. (D) Specificity of the ELISA based on human immunoglobulin G1 anti-DGP antibody UCD1002–1E03 used to detect deamidated peptide was confirmed by serial dilutions of native (biotin-GSGSGS-PLQPQQPFP) and deamidated (biotin-GSGSGS-PLQPEQPFP) gluten peptides. (E) Detection of shed cells in the gut lumen. Small-intestinal luminal content was collected in a ligated intestinal loop assay during 30 minutes, and cells were collected by flushing intestinal loops with phosphate-buffered saline/2% fetal calf serum. Enterocytes were identified as EpCAM+/CD45− and leukocytes as EpCAM−/CD45+ cells. Live cells were defined as 7-aminoactinomycin D (7-AAD)−/annexin V−, early apoptotic cells as 7-AAD−/annexin V+, and dead cells as 7-AAD+/annexin V+. EpCAM, epithelial cell adhesion molecule; FSC-A, forward scatter area; SSC-A, side scatter area. (F) Quantification of shed enterocytes with re [file NIHMS2077461-supplement-Supplementary_figure.jpg]
